# Gaucher-like Cells in Thalassemia Intermedia: Is It a Challenge?

**DOI:** 10.3390/diseases11040161

**Published:** 2023-11-06

**Authors:** Veroniki Komninaka, Pagona Flevari, Georgios Karkaletsis, Theodoros Androutsakos, Theofili Karkaletsi, Ioannis Ntanasis-Stathopoulos, Evaggelia-Eleni Ntelaki, Evangelos Terpos

**Affiliations:** 1 Centre of Excellence in Rare Haematological (Haemoglobinopathies) & Rare Metabolic (Gaucher Disease) Diseases, Laiko General Hospital, 11527 Athens, Greecefpagona@yahoo.gr (P.F.);; 2 Medical School, Otto von Guericke University Magdeburg (OVGU), 39120 Magdeburg, Germany; 3 Department of Pathophysiology, Medical School, National and Kapodistrian University of Athens, 11527 Athens, Greece; tandroutsak@med.uoa.gr; 4 Medical School, Charité—Berlin University of Medicine, 10117 Berlin, Germany; 5 Department of Clinical Therapeutics, Medical School, National and Kapodistrian University of Athens, 11528 Athens, Greece

**Keywords:** thalassemia intermedia, Gaucher-like cells, Gaucher cells, lysosomal storage disease, bone marrow

## Abstract

We describe two cases of thalassemia intermedia (TI) patients with the presence of Gaucher-like cells in hematopoietic tissue biopsies, raising diagnostic dilemmas. The first is a 56-year-old female with bone lesions, splenomegaly, hypochromic microcytic anemia and Gaucher-like cells in the bone marrow, with a final diagnosis of TI, and the second is a 69-year-old male with TI, monoclonal gammopathy of undetermined significance (MGUS) that accelerated to multiple myeloma (MM) requiring treatment, bone disease and Gaucher-like cells in the bone marrow and the spleen, and heterozygoty of Gaucher disease (GD). Gaucher-like cells are difficult to differentiate from true Gaucher cells, that are the hallmark of GD suspicion. These cells are usually reported in the lymphohematopoietic system. They have been described in myeloproliferative disorders, hematological malignancies, infectious diseases, hemoglobinopathies and other hemolytic anemias. The presence of Gaucher-like cells in patients with thalassemia major has been well documented, whereas there are limited references regarding cases with thalassemia intermedia. The identification of these cells in thalassemia probably reflects the high cell turnover. The bony complications in GD and TIare not yet fully explained in the literature, and this raises the question of whether Gaucher-like cells could play a pathogenetic role in the bone disease of thalassemia, as Gaucher cells are considered to play a similar role in bone complications of GD. Moreover, given the rarity and similarity of Gaucher and Gaucher-like cells, we would like to highlight that the presence of Gaucher-like cells in the bone marrow should not be overlooked, as they might be obscuring an underlying pathology, in order to ensure that hematologists, internists and hematopathologists will be promptly and accurately diagnosed.

## 1. Introduction

Gaucher disease (GD) is a hereditary lysosomal storage disease, caused by a deficiency in the activity of acid-b-glucosidase leading to glucosylceramide accumulation in tissue macrophages, giving rise to so-called ‘Gaucher cells’. Pseudo-Gaucher cells are macrophages scavenging membrane glycolipids in the setting of high cell turnover, and they have been described in various conditions, such as thalassemia. Thalassemias are a group of inherited disorders characterized by a decreased production of globin chains of haemoglobin and are widely heterogeneous, both genetically and clinically. The presence of Gaucher-like cells in patients with thalassemia major is known.

There are limited references in the literature regarding thalassemia intermedia (TI) and Gaucher-like cells. Given the rarity of this phenomenon, we would like to present our experience in order to enhance the knowledge in this field. Thus we describe two cases: a patient who was investigated for the presence of Gaucher-like cells in the bone marrow biopsy and was finally diagnosed with thalassemia intermedia, and secondly, a patient with thalassemia intermedia who developed multiple myeloma (MM) and presented Gaucher-like cells in the bone marrow and spleen biopsy.

### 1.1. Case 1

A 56-year-old female was under investigation due to a thoracic vertebra fracture, osteolytic lesions and osteoporosis under treatment with denosumab. A physical examination revealed only splenomegaly. A skeletal survey with a bone scan was positive for a diffused increased radioisotope uptake and an FDG PET-CT revealed a diffused FDG radiotracer uptake, suggesting the presence of a hematological condition such as multiple myeloma/low grade lymphoma or other benign conditions. A spinal computed tomography scan (CT) was also performed, confirming the presence of bone lesions and of a thoracic vertebra fracture. A bone marrow biopsy was performed in order to investigate the presence of multiple myeloma and revealed a medium infiltration (≤20% of the bone marrow cellular population) with Gaucher-like cells [PGM1+ (CD68 antibody), lysozyme+, PAS + (Periodic acid—Shiff stain)], suggesting the possible diagnosis of a Lysosomal Storage Disease (LSD); the erythroid lineage was hyperplastic, but no monoclonal plasmatocytes were identified (3% polyclonal plasmatocytes). In parallel, further investigations were carried out. Peripheral blood testing for bcr-abl and JAK2V617F mutations was negative, serum electrophoresis for monoclonal band was negative, serum and urine immunofixation were negative, urine testing for Bence jones proteins was also negative, serum free κ chains were 8.95 mg/L (normal reference values: 3.3–19.4), free λ chains were 8.83 mg/L (normal reference values: 5.71–26.3), free κ/λ ratio 1.01, serum creatinine was 0.51 mg/dL (normal reference values: 0.51–0.95), serum calcium was 9.7 mg/dL (normal reference values: 8.6–10.2), and β2 microglobulin was 1.66 mg/dL (normal reference values: 0.97–2.64). Serum immunoglobulins were measured within normal reference values. The complete blood count showed Hb of 10.5 g/dL, MCV of 59.5 fL, MCH of 18.8 pg, WBC count of 7.90 × 10^3^/uL, and PLT count of 180 × 10^3^/uL. Serum ferritin was 119 ng/mL and the Hb HPLC electrophoresis showed HbA2 6.5%, HbA 69.2% and HbF 17.5%, findings leading to the diagnosis of thalassemia intermedia. Genotype characterization revealed the presence of the -101 (C>t) beta+ (silent) mutation in compound heterozygosity with the Codon 39 (C>T);CAG(Gln)->TAG (stop codon) beta0 (HGVS name: c.[-151C>T];[118C>T] Reference Sequence NM_000518.5. Additionally, the serum chitotriosidase measurement was 52 nmol (within normal reference values) and at the tandem mass spectrometry from dried blood spot, the quantitative measurement of lyso-GL-1 was 10.9 ng/mL (below the cutoff value of 14 ng/mL), and the glucocerebrosidase and acid sphingomyelinase activities of the patient were above the cut-off value. Taking all the above together, Gaucher and Niemann-Pick diseases were considered unlikely diagnoses for the patient (therefore no molecular testing was performed), while the diagnosis of TI was established.

### 1.2. Case 2

**Figure 1:**
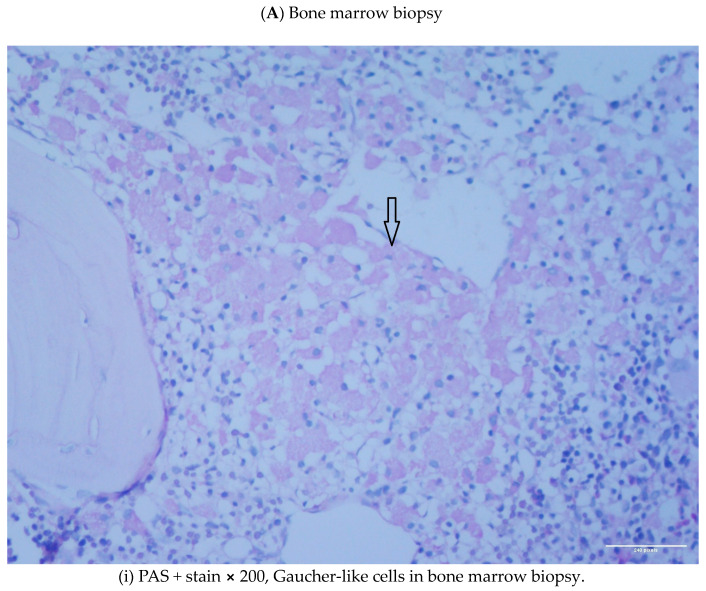

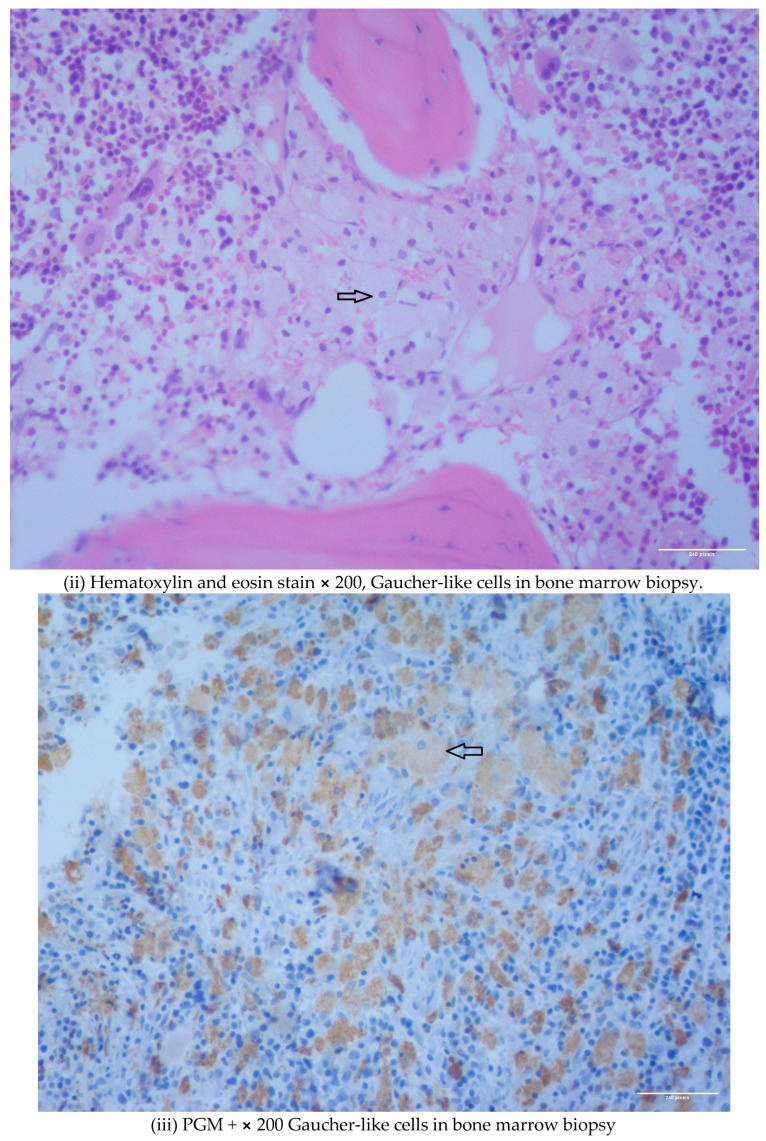

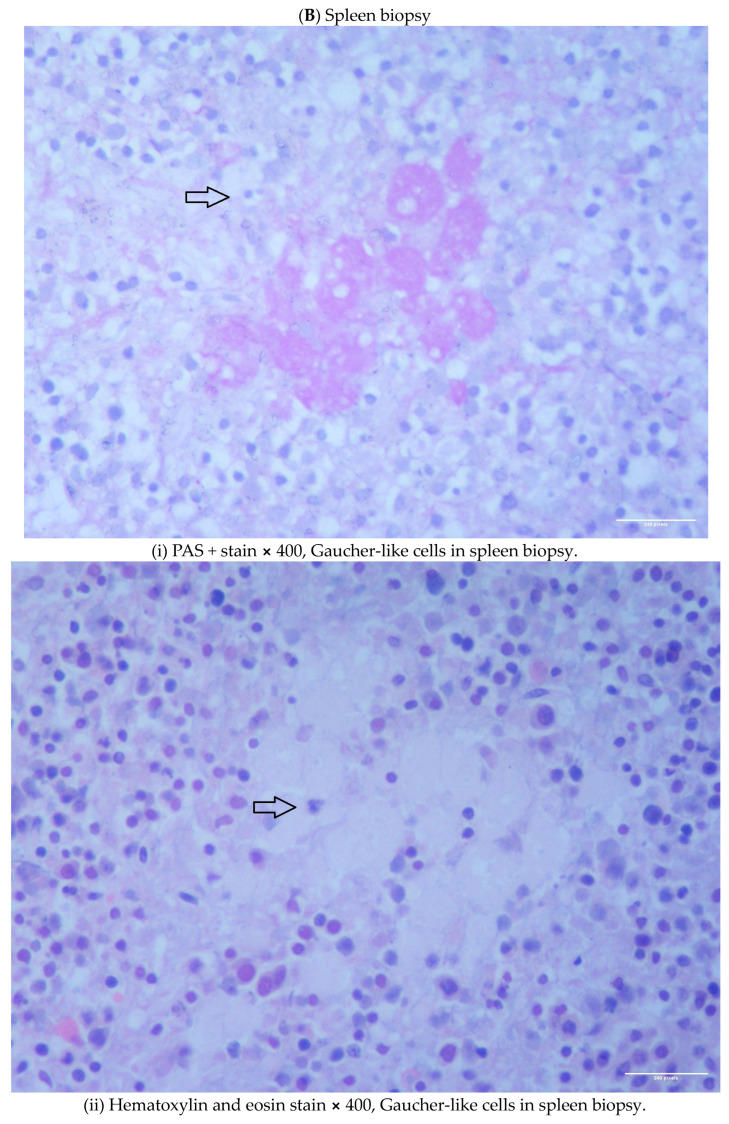

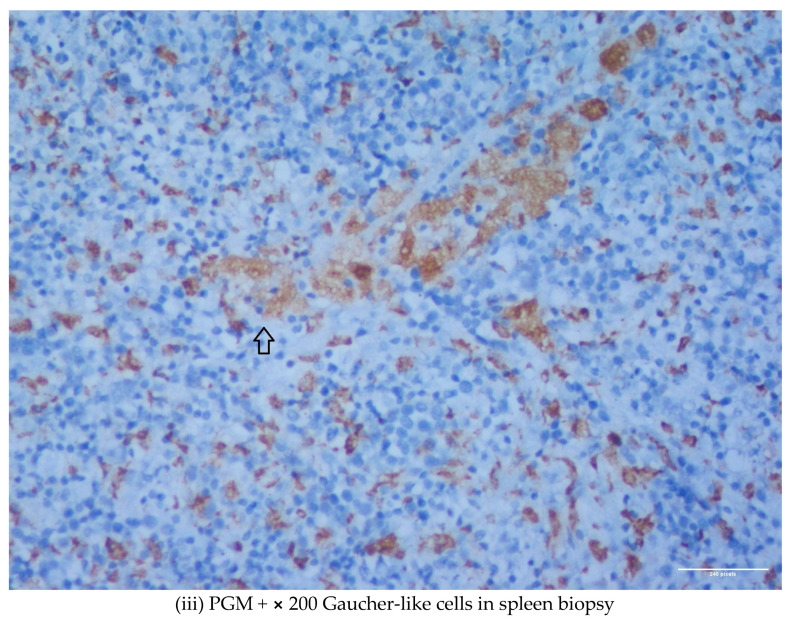
Case 2. Characteristic pathology images from (**A**) Bone marrow biopsy and (**B**) Spleen biopsy.

A 69-year-old male with a diagnosis of thalassemia intermedia and osteopenia followed up at our center since 1999. The hematological indices upon the diagnosis of TI were Hb 9.8 g/dL, Hct 29.5%, MCV 75.8 fL, and MCH 25.2 pg, and the Hb electrophoresis showed HbF 97.1% and HbA 2.9%. Genotype characterization for thalassemia was homozygosity for the Sicilian delta-beta thalassemia deletion (δβSic Sicilian (delta beta) 0-Thal, HGVS name NG_000007.3:g.64336_77738del13403), that is characterized by the absence of synthesis of the delta and beta globin chains with an increase of gamma chain synthesis. Due to positive serum electrophoresis for monoclonal band (IgGλ), a bone marrow biopsy was performed in 2014. The biopsy revealed a bone marrow infiltration of 20% by monoclonal plasma cells and the presence of Gaucher-like cells (PGM-1+) at a percentage of 25–30% (Figure 1A(i–iii)—Case 2), establishing the diagnosis of smoldering multiple myeloma and raising the suspicion of the co-existence of a lysosomal storage disease (Gaucher disease/Niemman-Pick). The whole-body low-dose CT was negative for lytic skeletal lesions, and regarding the MM, the patient remained under surveillance. In parallel, due to a hyperhemolysis syndrome, the patient underwent a splenectomy and the spleen biopsy revealed the presence of Gaucher-like cells (PGM-1+, PAS+) (Figure 1B(i–iii)—Case 2). A new bone marrow biopsy was performed a few months after the previous one and showed bone marrow infiltration of 30% by monoclonal plasma cells and the presence of Gaucher-like cells (PGM-1+, PAS+, Lysozyme+, TRAP-, CD1a-, CD138-), again raising the suspicion of a lysosomal storage disease coexistence, so further investigations were performed. Serum chitotriosidase activity had zero compatibility with 6% of the population with no chitotriosidase synthesis, and the β-glucosidase activity was of a value compatible with Gaucher carriers; therefore, no molecular testing was performed. Therefore, the diagnosis of Gaucher disease was not confirmed. In 2021, the patient’s anemia worsened, and they developed skeletal lytic lesions and elevated serum calcium. Treatment for multiple myeloma was initiated with lenalidomide and dexamethasone, and now the patient is in remission with lenalidomide maintenance therapy. A new bone marrow biopsy confirmed the multiple myeloma remission and once again, the presence of Gaucher-like cells (30% infiltration and same morphology features).

## 2. Discussion

The diagnosis of GD is suspected with the identification of Gaucher cells in bone marrow, liver and spleen biopsies. Gaucher cells are enlarged macrophages containing undigested glucocerebroside. Gaucher cells have small, usually eccentrically placed nuclei and cytoplasm with characteristic wrinkles or striations. These cells possess abundant eosinophilic wrinkled cytoplasm which is PAS positive and diastase resistant. Ultra-structurally, they contain distended lysosomal sacs filled with tightly packed twisted deposits. They have a distinct appearance similar to “wrinkled tissue paper” under light microscopy, because substrates build up within the lysosome [1,2]. Figure S1 shows Gaucher cells in a bone marrow smear with May Grunwald-Giemsa (MGG) stain. They infiltrate many organs including the bone marrow, spleen and the liver, leading to the clinical manifestations of Gaucher disease.

**Figure 2:**
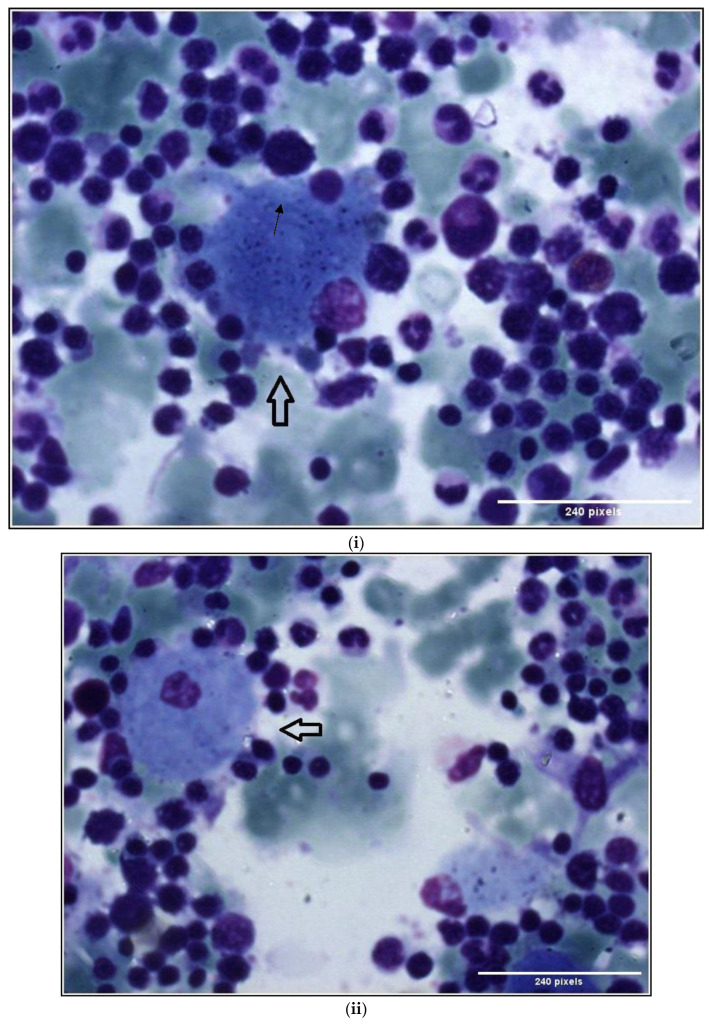

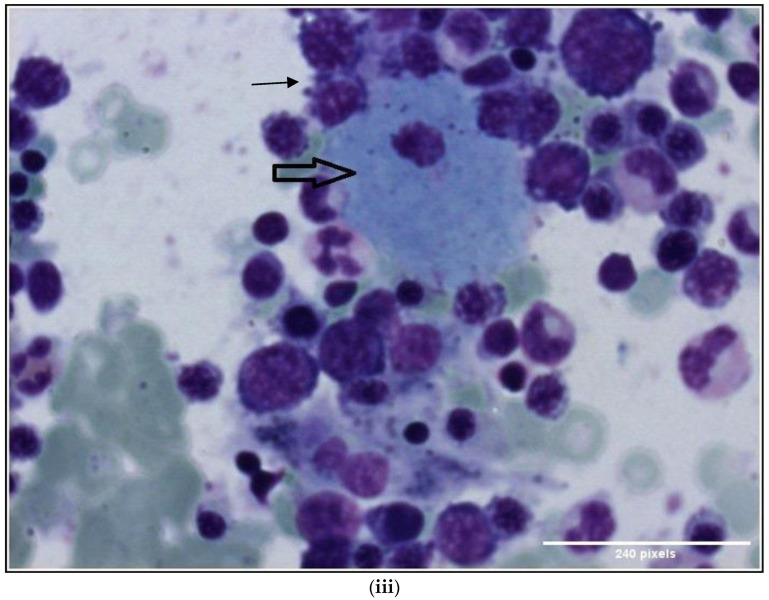
Case 1. Gaucher-like cells in bone marrow smear with May Grunwald-Giemsa (MGG) × 100 (**i**–**iii**).

Gaucher-like cells are bone marrow macrophages (histiocytes) with rounded, blue, lamellar cytoplasm resembling “onion skin” and are considered to be related to increased cell turnover. Although these cells show a superficial resemblance to Gaucher cells under light microscopy, ultra-structurally their parallel intralysosomal deposits lack the twisted nature characteristically found in Gaucher cells [3]. The Gaucher-like cells in thalassemia typically contain bundles of fine fibrils [4]. Figure 2i–iii—Case 1. Bone marrow smears show Gaucher-like cells with the use of May Grunwald-Giemsa (MGG) stain. They have been described in various hemolytic and myeloproliferative disorders, in hematologic malignancies and in infectious diseases such as hemoglobinopathies, some subtypes of congenital dyserythropoietic anemia, chronic myeloid leukemia, Waldenstrom macroglobulinemia, multiple myeloma, Hodgkin’s lymphoma and tuberculosis [5,6,7,8,9,10,11,12,13,14].

Gaucher-like cells are difficult to differentiate from true Gaucher cells using morphology alone; cytochemistry and electron microscopic findings aid in definitive differentiation. Iron staining should be performed in order to differentiate Gaucher-like cells from true Gaucher cells. This method is diagnostic because Gaucher cells have diffused iron staining, whereas Gaucher-like cells do not. Additionally, the ultrastructure of the crystalline inclusions on electron microscopic examination may also help to distinguish these two types of cells, Gaucher-like cells from true Gaucher cells, since Gaucher-like cells do not contain typical tubular cytoplasmic inclusions, which are present in Gaucher cells [9,15,16]. Upon the identification of Gaucher-like cells in hematopoietic biopsies, iron staining as a routine method could be an important and feasible diagnostic tool.

A previous study by Beltrami et al. [4] suggested that the Gaucher-like cells in the spleen and marrow of thalassemic patients developed from histiocytes that were unable to metabolize completely the products of the phagocytosed blood cells. They could identify transitional cells containing both fine fibrillary material and ingested blood cells (leukocytes, red blood cells, platelets and plasma cells). Beltrami et al. reported that the histiocytes in thalassemia have a distinctive morphology when visualized using an electron microscope; they are typically filled with cytoplasmic inclusion bodies that contain fine fibrils arranged in parallel. Ishihara et al. [17] suggested that for Gaucher-like cells in general, there might be an unidentified underlying functional abnormality in the histiocytes.

The presence of Gaucher-like cells in the bone marrow and the spleen of patients with thalassemia major has been well documented [4,18]. In other cases of thalassemia intermedia with high bone marrow turnover and an absence of clinical signs of a storage disorder, a diagnosis of Gaucher-like cells was also given [7,19]. These cells have not been reported to occur outside the lymphohematopoietic system. In our cases, Gaucher-like cells were identified in the bone marrow and the spleen. The presence of Gaucher-like cells in other locations is extremely rare, but there is a reference regarding their presence in the periosteum [20]. A hypothesis could involve their migration from the bone marrow across the cortical bone to the periosteum. Another interpretation is that these aggregates represent a generalized Gaucher-like histiocytic response, a process that could explain similar clustering of these cells in other parts of the body.

Regarding the above-mentioned cases, both of our thalassemia patients presented with bone disease and bone marrow infiltration of Gaucher-like cells. Up to date, the pathophysiology of bone complications in GD is not adequately clarified. According to the literature, the infiltration of Gaucher cells in the bone marrow cavity may be related to the following situations: firstly, the interference with vascularity at the cortical surface through red marrow expansion, and secondly, the changes in cytokine expression caused by macrophage activation from glucocerebroside accumulation [21,22,23].

In thalassemia, osteoporosis is emerging as a significant problem and its etiology is considered to be multifactorial. Bone marrow expansion, iron overload, multiple endocrinopathies, iron deposit in the skeleton, deferoxamine bone toxicity, vitamin D deficiency and altered cytokines network play an important role in bone damage [24,25,26]. The altered cytokines network (interleukins: ILs, tumor necrosis factor α, receptor activator of NF-κB: RANK and its soluble ligand RANKL, osteoprotegerin: OPG) in thalassemic patients is considered responsible for osteoclasts activation [27].

Under the same terms, in cases of activated macrophages resulting in altered cytokine mechanisms and bone disease, such as Langerhans cell histiocytosis (LCH), the bone involvement is usually presented as osteolytic lesions with low bone mineral density. Cytokine production in aggregates of such histiocytes/macrophages increases bone turnover and accelerates the rate of bone loss, as occurs in LCH lesions [28].

Given these references, could Gaucher-like cells play a pathogenetic role in bone lesions and also in thalassemia? Can these cells also be considered as activated macrophages resulting in changes in cytokine expression and participating in bone disease of thalassemia patients, like Gaucher cells? This hypothesis could lead to more investigation and may add more information to the puzzle of pathogenesis of thalassemia bone disease. For example, it would be interesting to study the cytokines network profile and the bone disease status in a population of thalassemia patients with no deposits of Gaucher-like cells in hematopoietic tissues in comparison with a population of thalassemia patients with presence of Gaucher-like cells in such tissues. Unfortunately, our study has several limitations and cannot address these issues; the generalizability of our findings should be made with caution since we have included only two cases. In this context, no robust statistical analyses with adequate statistical power could be made in order to reach firm conclusions. Further prospective research with larger sample sizes and quantitative data is necessary to corroborate the proposed hypotheses and solidify the conclusions.

Multifocal large aggregates of Gaucher-like cells are rarely reported and can create potential confusion with storage cells of Gaucher type. The presence of Gaucher-like cells in bone marrow should be dealt with caution. Given the rarity of Gaucher and Gaucher-like cells, hematology clinicians and internists should be aware not only of the potential for Gaucher disease, but also of the need to consider and evaluate the finding of Gaucher-like cells in their differential diagnosis. It is important for hematologists and hematopathologists to be aware of such a condition in order to make a prompt and accurate diagnosis [29]. For example, our thalassemia intermedia patient in the second case was diagnosed with multiple myeloma. Gaucher disease may co-exist in patients with monoclonal gammopathies including multiple myeloma [30,31,32].

There is a reference in the literature regarding a single clinical case in which the percentage of Gaucher-like cells in a thalassemia patient decreased during treatment with low-dose thalidomide due to hyperhemolysis syndrome and alloimmunization [33]. In our second case, the percentage and the morphology of Gaucher-like cells in the bone marrow biopsies were not impacted by the chemotherapy administration, despite the fact that patient reached remission. Although Gaucher-like cells are often present in malignancies, their exact role regarding the pathogenesis, clinical course and therapy response remains unclear, and more research is needed.

There is another reference noting that the presence of Gaucher-like cells should be taken under consideration in splenectomized thalassemia patients with inappropriately low reticulocytes and increased blood transfusion needs [34]. Our little experience on the above-mentioned cases and all these references in the literature raise suspicion of the general pathogenetic role of these cells and more investigations may enlighten it.

Nevertheless, in daily practice, the presence of Gaucher-like cells in the bone marrow might be obscuring an underlying pathology, so we would like to point out that this finding should not be overlooked. In order to make a final and precise diagnosis, clinicians should be aware of possible associations, appropriate immunohistochemistry and relevant additional investigations according to clinical findings.

## 3. Conclusions

Gaucher-like cells have been described in various conditions with high cell turnover, such as in major and intermedia thalassemia. The presence of Gaucher-like cells probably reflects the increased load of leukocyte membrane-derived glucosylceramide in macrophages. The normal mechanisms/pathways for its removal may be saturated and overloaded, resulting in an acquired phenotype resembling enzyme deficiency. The sheet-like pattern of Gaucher-like cells may obscure the underlying primary pathology causing diagnostic difficulties, so all involved professionals should include this knowledge in their differential diagnosis.

## Data Availability

The data presented in the manuscript are available from the corresponding author upon reasonable request.

## Supplementary Materials

The following supporting information can be downloaded at: https://www.mdpi.com/article/10.3390/diseases11040161/s1, Figure S1: Gaucher cells in bone marrow smear with May Grunwald-Giemsa (MGG) stain × 100.
