# Supplementary material for: Gaucher-like Cells in Thalassemia Intermedia: Is It a Challenge?
Source: Diseases. 2023 Nov 6;11(4):161. doi: 10.3390/diseases11040161 (PMC10660717; doi:10.3390/diseases11040161)

diseases

Figure S1. Gaucher cells in bone marrow smear with May Grunwald-Giemsa (MGG) stain x 100

(from authors' records)

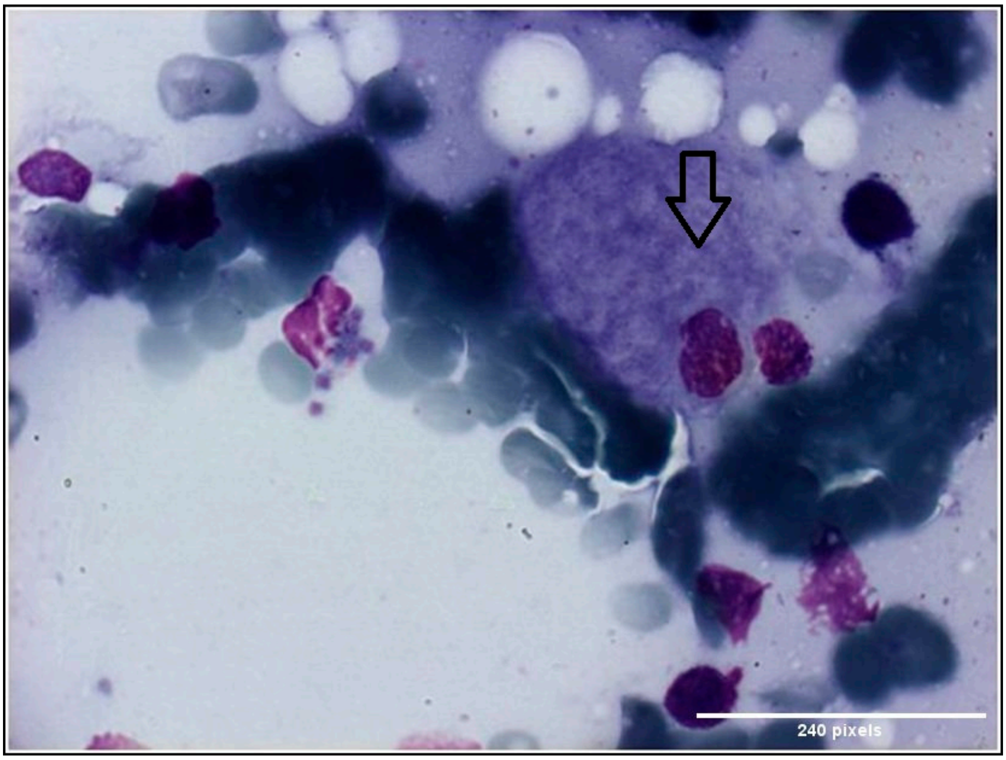

Supplement: Supplementary file 1 [file diseases-11-00161-s001.zip › diseases-2677692-supplementary.pdf]
